# Temozolomide Is a Potential Therapeutic Tool for Patients With Metastatic Pheochromocytoma/Paraganglioma—Case Report and Review of the Literature

**DOI:** 10.3389/fendo.2020.00061

**Published:** 2020-02-18

**Authors:** Anli Tong, Ming Li, Yunying Cui, Xiaosen Ma, Huiping Wang, Yuxiu Li

**Affiliations:** ^1^Department of Endocrinology, Key Laboratory of Endocrinology, National Health Commission of the People's Republic of China, Peking Union Medical College Hospital, Peking Union Medical College, Chinese Academy of Medical Sciences, Beijing, China; ^2^Department of Clinical Laboratory, Peking Union Medical College Hospital, Peking Union Medical College, Chinese Academy of Medical Sciences, Beijing, China

**Keywords:** metastatic pheochromocytoma/paraganglioma, therapy, temozolomide, DNA Methylation, SDHB

## Abstract

**Context:** Metastatic pheochromocytoma/paraganglioma (MPP) therapy mainly involves radionuclide therapy, chemotherapy, and targeted therapy. In recent years, temozolomide (TMZ) showed great promise in some MMP patients, especially those with *SDHB* germline mutation. We reported a patient with MPP who did not have any known germline genetic change and responded remarkably well to TMZ monotherapy.

**Case presentation:** The patient was a 41-year-old woman with local and distant recurrence (soft tissues and bone metastases) of retroperitoneal paraganglioma. She suffered from dizziness, palpitation, sweating, weight loss and constipation, with the blood pressure fluctuating substantially from 130/100 mmHg to 190/120 mmHg, although she was on phenoxybenzamine and metoprolol medication. The patient showed clinical and radiological response after 3-cycle TMZ therapy. Upon 15 cycles of TMZ therapy, her symptoms were dramatically alleviated, urinary norepinephrine excretion decreased from 1,840 μg/24 h to 206 μg/24 h, and CT showed that the lesions further shrank. Molecular profiling of the tumor tissue of the patient revealed hypermethylation of the O6-methylguanine-DNA-methyltransferase (*MGMT*) promoter and a negative immunostaining for MGMT. Globally, only 26 cases of MPP treated with TMZ have been described so far. TMZ is effective, especially in patients with *SDHB* mutation, which can be explained by the silencing of MGMT expression as a consequence of *MGMT* promoter hypermethylation in *SDHB*-mutated tumors. Although, in general, patients with *SDHB* mutation or MGMT promoter hypermethylation have better response to TMZ, there are also exceptions. Severe side effects are uncommon, with only 17.4% patients experiencing Grade 3 toxicities, including lymphopenia, and hypertension.

**Conclusions:** TMZ is effective and safe in MPP patients, and, it may work better on patients with *SDHB*-related MPP. Measurement of MGMT expression might help assess the tumor sensitivity to TMZ but this needs further systematic investigation.

## Introduction

Pheochromocytoma (PHEO) and paraganglioma (PGL) are rare tumors originating from adrenal medulla, sympathetic paravertebral ganglia, and parasympathetic ganglia. Metastatic pheochromocytoma/paraganglioma (MPP), defined as metastasis in non-chromophilic tissues, such as lymph nodes, bone, liver, lung, etc, accounts for 10–17% of all PHEO/PGL. Metastasis in MPP, mostly multiple and invasive, usually could not be completely resected, and the mean overall 5-year survival rate of the patients is 44% (1).

The first and most important treatment for MPP is surgery. But for the non-resectable tumors and metastatic loci, second line therapy includes systemic treatment, such as radionuclide therapy, chemotherapy and targeted therapy, and local treatment, such as radiotherapy, ablation and embolization. Radionuclide therapy includes ^131^I-MIBG therapy and peptide receptor radionuclide therapy (PRRT, such as 177Lu-DOTATATE), which can only be used for the treatment of tumors with uptake of the tracer (2, 3). Molecular targeted therapy, such as sunitinib, has shown great potential in the treatment of MPP, but needs to be further investigated (4). Chemotherapy mainly includes CVD regimen (cyclophosphamide, vincristine, and dacarbazine) and temozolomide (TMZ) (5, 6). CVD is the most commonly used chemotherapeutic regimen for MPP (7), and TMZ, an oral alkylation chemotherapeutic agent, was first reported to be used for the treatment of MPP in 2006 (8). Because MPP is relatively rare, so far, only 26 cases of MPP were reportedly treated with TMZ, most of them being presented in the form of case reports (8–15). TMZ was effective in MPP patients, especially those with *SDHB* germline mutation (12). Although in glioblastoma, the efficacy of TMZ is strongly related to the level of O^6^-methylguanine-DNA methyltransferase (*MGMT*) promoter hypermethylation (16), it is not clear whether a similar relationship exists in MPP.

In this report, we presented a Chinese MPP patient who responded fairly well to TMZ monotherapy. This patient did not have any known germline genetic change. The efficacy and safety of TMZ therapy in MPP were also discussed at length in the paper.

## Case Presentation

In June 2018, a 41-year-old woman presented to the Peking Union Medical College Hospital, Beijing, China. In 2015, she had episodes of headache, palpitation, and sweating. Her blood pressure was 200/120 mmHg. Imaging studies revealed a retroperitoneal mass of about 8 cm in size. In November 2015, she underwent surgical resection and the mass was pathologically diagnosed as PGL. Postoperatively, her symptoms, and hypertension were relieved.

Two years later, her blood pressure again rose to 150–160/100 mmHg. The patient was found to have multifocal bone lesions on contrast-enhanced computed tomography (CT) and ^18^F-fluorodeoxyglucose positron emission tomography/computed tomography (^18^F-FDG-PET/CT). In March 2018, the patients underwent ^131^I-meta-iodo-benzyl-guanidine (^131^I-MIBG) therapy at a dose of 200 mCi. Post-treatment scintigraphy suggested uptake of ^131^I-MIBG to most metastatic lesions was unsatisfactory.

**Figure 1:**
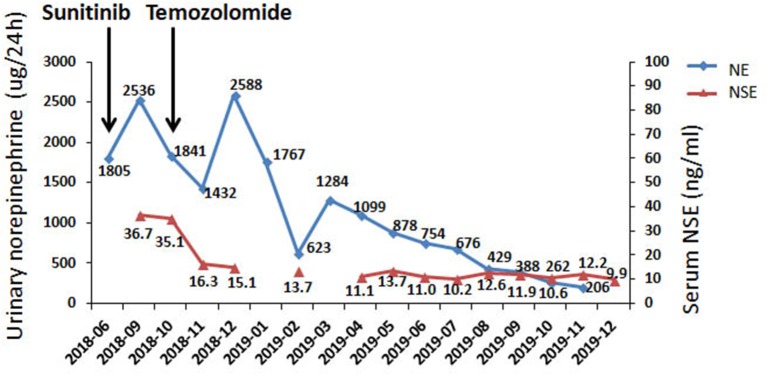
Biochemical responses of the patient to TMZ therapy. NE, norepinephrine; NSE neuronspecific enolase.

**Figure 2:**
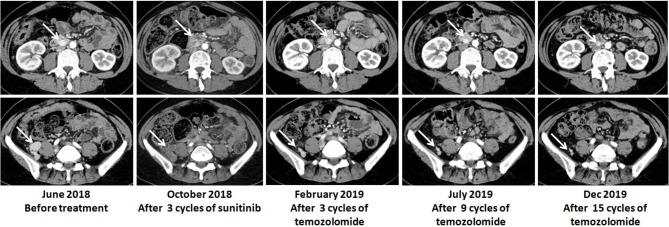
Radiological responses of the patient to TMZ therapy.

In June 2018, the patient was referred to our hospital. She suffered from dizziness, palpitation, sweating, weight loss and constipation, although she was on phenoxybenzamine 20 mg q8h and metoprolol 25 mg tid. The blood pressure fluctuated greatly from 130/100 to 190/120 mmHg. Urinary norepinephrine excretion was 1,804 μg/24 h [reference value (r.v.): 17–41 μg/24 h], urinary epinephrine was 4 μg/24 h (r.v.: 2–6 μg/24 h), and dopamine was 203 μg/24 h (r.v.: 121–331 μg/24 h) (Figure 1). CT scan showed multiple high-intensity nodules below and latero-posterior to right kidney and below and posterior to duodenal horizontal segment (Figure 2). Multiple bony defects were found in lumbar vertebrae, sacrum and iliac bones, suggesting metastases. She was put on sunitinib at 37.5 mg daily from July 2018. After 3 months of treatment, her hypertension deteriorated, with blood pressure being 180/120 mmHg even at 100 mg of phenoxybenzamine and 75 mg of metoprolol on daily basis. Her urinary catecholamine did not decrease, with norepinephrine excretion being 1,840 μg/24 h, epinephrine 5 μg/24 h and dopamine 258 μg/24 h. Serum neuron-specific enolase (NSE) was 35.1 ng/ml (r.v.: 0–16.0 ng/ml) (Figure 1). CT showed that some of the aforementioned lesions enlarged (Figure 2).

In Oct 2018, the patient was put on TMZ at 150 mg/m^2^/d on days 1–5 in a 28-day cycle. In the second and the following cycles, she took TMZ at 200 mg/m^2^/d (on a 5/28-day regimen). After three cycles, blood pressure of the patient dropped significantly, and CT suggested that the lesions were significantly diminished (Figure 2). After nine cycles, her symptoms, including dizziness, palpitation, sweating and constipation, were dramatically alleviated, and at reduced dose of phenylbenzylamine (10 mg q8h) and metoprolol (25 mg bid), her blood pressure was lowered to 130-150/90-100 mmHg. After fifteen cycles, her blood pressure decreased further and was 120-130/70-80 mmHg on phenylbenzylamine (5 mg q12h) and metoprolol (12.5 mg bid). Urinary norepinephrine excretion decreased to 206 μg/24 h. Urinary epinephrine and dopamine were 2 and 281 μg/24 h, respectively (Figure 1). CT showed that the lesions further shrank (Figure 2). The patient well-tolerated TMZ and only suffered from mild nausea on the first day of the medication. No bone marrow suppression was observed. Written informed consent for data publication was obtained from the patient.

### Genetic Detection

Genetic screening of the genomic DNA from peripheral blood leukocytes of the patient was conducted by using targeted next-generation sequencing (NGS) that involved 20 genes (*NF1, VHL, RET, SDHA, SDHB, SDHC, SDHD, SDHAF2, MAX, TMEM127, FH, KIF1B, BAP1, IDH1, EPAS1, EGLN2, EGLN1, EGLN3, HRAS, MDH2*), and the sequencing failed to identify any pathogenic genetic change (Supplement 1). Large deletions of *SDHB, SDHC, SDHD, SDHAF2*, and *VHL* were excluded by MLPA using a MLPA reagent kit (MRC-Holland) (Supplement 2).

### Pathological Features and Immunohistochemistry

**Figure 3:**
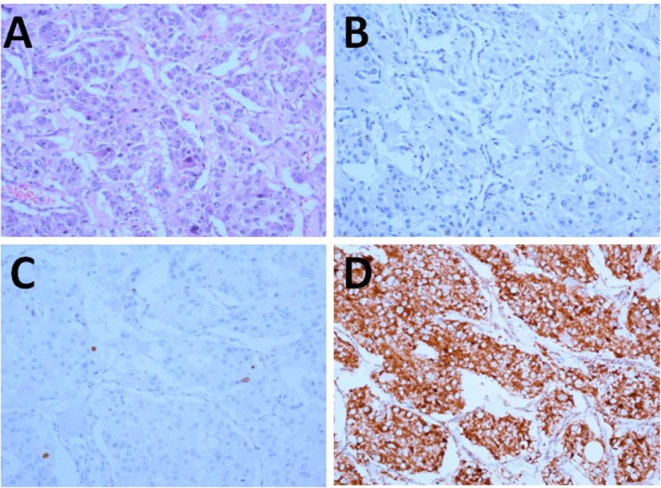
Pathology and immunohistochemistry of the tumor (x200). **(A)** HE; **(B–D)** Immunohistochemical staining for MGMT **(B)**, Ki-67 **(C)**, and SDHB **(D)**.

Hematoxylin-eosin staining showed that the tumor cells were arranged in nest-like pattern, having basophil cytoplasm, and large nuclei. The tumor tissues were immunohistochemically examined by using the EnVision detection kit and was found to be negative for MGMT and positive for SDHB, with a Ki-67 index of 1% (Figure 3).

### *MGMT* Methylation Analysis

Methylation of four CpG sites in exon 1 of the human *MGMT* gene was quantitatively determined by employing the *MGMT* Pyrosequencing, and the results showed that the four sites were hypermethylated (Supplement 2).

## Discussion

MPP accounted for 10–17% in all PHEO/PGL cases. Compared with PHEO, PGL is more prone to metastasis. Five to twenty percent of PHEO and 15–35% of sympathetic PGL metastasize (17). Thirty five to forty percent of patients with PHEO/PGL have germline genetic mutation, and the rate is 50% in patients with MPP. So far, over twenty genes associated with PGL hereditary syndromes have been reported. Mutations in *SDHB* are one of the most common causes of familial PHEO/PGL, and tend to be associated with MPPs, particularly MPPs that afflict younger patients (18, 19). The patient in our study did not have any known germline genetic change, including such genes related to MPP as *SDHB* and *FH*.

TMZ is widely used for the treatment of various tumors, such as adult refractory pleomorphic glioblastoma, anaplastic astrocytoma, pancreatic neuroendocrine neoplasm and invasive pituitary tumor and pituitary cancer (16, 20, 21). The cytotoxic activities of TMZ lies in DNA methylation and failure of mismatch repair (22, 23). TMZ resistance is mediated predominantly by MGMT protein. MGMT is a highly-efficient DNA repair enzyme, which can repair methylated DNA. Tumor cells lacking MGMT expression are significantly more sensitive to the cytotoxic effects of TMZ than their counterparts expressing functional MGMT (22, 23).

Only 26 patients have been reported to be treated by TMZ since 2006 (8–15). The two earliest studies examined MPP and other neuroendocrine tumors at the same time, and they didn't elaborate on the therapeutic effect of TMZ in MPP patients (8, 9). Therefore, in this paper, we reviewed the efficacy and safety of TMZ therapy in 23 MPP patients whose data were relatively complete (Table 1) (10–15). Except a retrospective study involving 15 patients with progressive MPP treated with TMZ, reported by Hadoux et al. (12), all the others were case reports. Most patients, just like our patient, received TMZ therapy at a dose of 150–200 mg/m^2^ (orally, once daily) on days 1–5 and the medication was repeated every 28 days, as it is used for glioblastoma patients. Two patients were given TMZ at a dose of 75 mg/m^2^/d for 3 weeks and were taken off the treatment for one week (21/28-day regimen) (13, 14). The total dosages of the two strategies were similar in each treatment cycle.

**Table 1:** Summary of the efficacy and safety of TMZ in the treatment of MPP.

**Pt**	**Sex/** **age**	**Gene mutation**	**MGMT methylation**	**MGMT expression**	**Location of mass**	**Prior treatment/** **PFS (mo)/** **effect**	**TMZ doses/** **cycles**	**Combination of other therapy**	**CgA response**	**MNs response**	**RECIST 1.1**	**PERCIST 1.0**	**Duration of TMZ**	**Side effect**	**References**
1	M/60	SDHB	Yes	NA	Bone and soft tissue metastases	Sunitinib/2/ PD Lanreotide+ CVD/6/PD	75 mg/m^2^ (21/28)/17 75 mg/m^2^ (21/35)/10	Lanreotide 120 mg q2w Zoledronic acid 4 mg q4w	Stable	Response^h^	NA	PMR	27 cycles	Grade 3 lymphopenia	(14)
2	F/46	SDHB	Yes	NA	Bone and soft tissue metastases	CVD/8/PD Lanreotide/ 15/PD	200 mg/m^2^ (5/28)/4 75 mg/m^2^ (21/28)/7	Capecitabine 750 mg/m^2^ bid (14/28)/4 Denosumab 120 mg+lanreotide 120 mg q2w/7	Response Normal^f^	NA	NA	PMR	13 cycles	No side effect	(14)
3	M/51	ND^b^	Yes	NA	Recurrence of PCC, liver and bone metastases	Sunitinib/ 4/PD	150 mg/m^2^ (5/28)	No	NA	Response Normal^f^	PR	NA	24 cycles	Grade 2 thrombocytopenia	(15)
4	F/54	MAX	NA	NA	Unresectable PGL	Sunitinib/4^d^	150 mg/m^2^ (5/28)	No	NA	Stable	SD	NA	23 cycles	No grade 3–4 side effects	(15)
5	M/53	SDHA	NA	NA	Bone metastases	Lanreotide+ denosumab/ 18/PD	75 mg/m^2^/d (21/28)	Propranolol 3 mg/kg/d	Decrease^g^	Decrease^g^	NA	PMR	14 cycles	Lymphopenia and thrombopenia	(13)
6	M/57	SDHB	Yes	NA	Lymph nodes and lung metastases	Gemcitabine+ oxaliplatin/8^d^ sunitinib/1/not assessed	200 mg/m^2^ (5/28)	Capecitabine 750 mg/m^2^ bid (14/28)	NA	NA	PR	NA	8 cycles	NA	(11)
7	F/60	NA	NA	NA	PGL, liver and lymph nodes metastases	No	250 mg/d (5/28)^e^	No	NA	Response^h^	PR	NA	5 cycles	Grade 1 nausea	(10)
8	NA^a^	SDHB	NA	Intact^c^	NA^k^	No	150 mg/m^2^ (5/28)/1 200 mg/m^2^ (5/28)	No	Response Normal^f^	Baseline MNs < ULN^j^	NA	PMR	30 months	NA^l^	(12)
9	NA^a^	SDHB	NA	NA	NA^k^	No	150 mg/m^2^ (5/28)/1 200 mg/m^2^ (5/28)	No	Stable	Stable	SD	PMD	3 months	NA^l^	(12)
10	NA^a^	SDHB	NA	Deficient^c^	NA^k^	No	150 mg/m^2^ (5/28)/1 200 mg/m^2^ (5/28)	No	Response Normal^f^	NA	PR	PMR	13 months	NA^l^	(12)
11	NA^a^	SDHB	NA	Deficient^c^	NA^k^	No	150 mg/m^2^ (5/28)/1 200 mg/m^2^ (5/28)	No	Response^h^	Baseline MNs < ULN^j^	SD	SMD	8 months	NA^l^	(12)
12	NA^a^	SDHB	NA	Deficient^c^	NA^k^	No	150 mg/m^2^ (5/28)/1 200 mg/m^2^ (5/28)	No	Baseline CgA <2ULN^j^	Response Normal^f^	PR	PMR	26 months	NA^l^	(12)
13	NA^a^	SDHB	NA	Deficient^c^	NA^k^	No	150 mg/m^2^ (5/28)/1 200 mg/m^2^ (5/28)	No	Response Normal^f^	Baseline MNs < ULN^j^	PR	PMR	21 months	NA^l^	(12)
14	NA^a^	SDHB	NA	NA	NA^k^	No	150 mg/m^2^ (5/28)/1 200 mg/m^2^ (5/28)	No	Progression^i^	Progression^i^	PD	PMD	3 months	NA^l^	(12)
15	NA^a^	SDHB	NA	Deficient^c^	NA^k^	No	150 mg/m^2^ (5/28)/1 200 mg/m^2^ (5/28)	No	Baseline CgA <2 ULN^j^	Response Normal^f^	PR	PMR	22 months	NA^l^	(12)
16	NA^a^	SDHB	NA	Intact^c^	NA^k^	No	150 mg/m^2^ (5/28)/1 200 mg/m^2^ (5/28)	No	Response^h^	Stable	SD	SMD	7 months	NA^l^	(12)
17	NA^a^	SDHB	NA	NA	NA^k^	No	150 mg/m^2^ (5/28)/1 200 mg/m^2^ (5/28)	No	NA	NA	NA	SMD	16 months	NA^l^	(12)
18	NA^a^	ND^b^	NA	NA	NA^k^	No	150 mg/m^2^ (5/28)/1 200 mg/m^2^ (5/28)	No	Stable	Stable	NA	PMD	3 months	NA^l^	(12)
19	NA^a^	ND^b^	NA	Intact^c^	NA^k^	No	150 mg/m^2^ (5/28)/1 200 mg/m^2^ (5/28)	No	Baseline CgA <2 ULN^j^	Stable	SD	PMD	9 months	NA^l^	(12)
20	NA^a^	ND^b^	NA	Intact^c^	NA^k^	No	150 mg/m^2^ (5/28)/1 200 mg/m^2^ (5/28)	No	Progression^i^	Progression^i^	SD	NA	14 months	NA^l^	(12)
21	NA^a^	ND^b^	NA	Intact^c^	NA^k^	No	150 mg/m^2^ (5/28)/1 200 mg/m^2^ (5/28)	No	Stable	NA	SD	PMD	4 months	NA^l^	(12)
22	NA^a^	ND^b^	NA	NA	NA^k^	No	150 mg/m^2^ (5/28)/1 200 mg/m^2^ (5/28)	No	NA	NA	NA	NA	1 months	NA^l^	(12)
23	F/41	ND	Yes	Deficient	Soft tissues and bone metastases	Sunitinib/ 3/PD	150 mg/m^2^ (5/28)/1 200 mg/m^2^ (5/28)	No	NA	Response^h^	PR	NA	15 cycles	Grade 1 nausea	This case

a *Median age of the 15 patients is 42.6 years old (range: 26–81), including 12 male and 3 female*.

b *Not found gene mutation of SDHB, SDHD, SDHC, SDHA, VHL, NF1, RET, TMEM127, and MAX*.

c *MGMT-intact tumors were defined by the presence of more than 20% positive tumor cells. MGMT deficient tumors were defined by the complete absence of positive tumor cells or by the presence of <20% positive cells*.

d *The patient stopped the therapy due to side effect*.

e *The patient weighed 115lb*.

f *Complete response with normalization of CgA or MNs*.

g *The detail data are not available from the original paper*.

h *Response was defined as a CgA or MNs concentration following treatment that was 50% lower than that of baseline concentration*.

i *Progression was defined as a CgA or MNs concentration following treatment that was 50% higher than that of baseline concentration*.

j *CgA <2 ULN or MNs < ULN is regarded as normal. And the biochemical response was not assessed in this case*.

k *The 15 patients had bone (12 cases), lymph nodes (11 cases), liver (6 cases), and lung metastases (5 cases)*.

l *Three out of the 15 patients experienced Grade 3 toxicities, including lymphopenia (2 cases) and hypertension (1 case). Grade 1 or 2 toxicities were digestive problems (8 cases), anaemia (7 cases), asthenia (7 cases), and constipation (3 cases)*.

*PR, Partial response; SD, Stable Disease; PD, Progressive Disease; PMR, Partial Metabolic Response; SMD, Stable Metabolic Disease; PMD, Progressive Metabolic Disease; NA, Not available; ND, Not found; ULN, Upper normal limit; PCC, Pheochromocytoma; PGL, Paraganglioma; TMZ, Temozolomide*.

Hadou et al. found that, of 15 MPP patients in their series on TMZ therapy, 5 (33%) achieved partial response (PR), 7 (47%) had stable disease (SD) and 3 (20%) suffered from progressive disease (PD). Ten patients had a *SDHB* germline mutation. Of these 10 patients, 9 patients were PR + SD and only 1 was PD. PFS was significantly longer in patients with *SDHB* mutation than in patients without mutations (19.7 vs. 2.9 months), 80% of PR patients exhibited MGMT deficience (12). In a separate cohort involving 190 PHEO/PGLs, *SDHB* mutation was associated with hypermethylation of the *MGMT* promoter and low expression of MGMT (12). This study demonstrated that TMZ was an effective antitumour agent for patients with *SDHB*-related MPP. And the result can be explained by the silencing of MGMT expression as a consequence of *MGMT* promoter hypermethylation in *SDHB*-mutated tumors (12). However, some other studies showed that, like our case, TMZ was also effective in patients with non-*SDHB* mutations. Tumors carrying mutations in Krebs cycle-related genes, such as *SDHx* and *FH*, exhibit CpG island hypermethylation, which was caused by the accumulation of specific metabolites. These metabolites lead to inactivation of DNA demethylases and thus to epigenetic alterations in the genome which leads to global gene expression changes, including loss of MGMT expression (24, 25). Our patient did not have any known germline pathogenic genetic change, but showed *MGMT* promoter hypermethylation, suggesting that other factors might also contribute DNA hypermethylation. Since the lesions of our patient shrank dramatically after TMZ treatment, we were led to speculate that *MGMT* promoter hypermethylation is ubiquitous in tumor tissues, and the factors causing hypermethylation exist in the early stage of the tumor development.

MGMT was expressed in tumor tissue of a patient with *SDHB* germline mutation (patient 8 in Table 1) (12). This patient had a PR for 30 months on TMZ, which suggested that TMZ might be effective for some patients even if MGMT was intact. In contrast, in a patient with both *SDHB* mutation and *MGMT* promoter hypermethylation (patient 6), the partial response to TMZ lasted only 8 month (11). Other two patients with *SDHB* mutation (patient 9 and patient 14) even had PD after TMZ treatment (12). These cases suggest that assessing the response to TMZ is very complex. And there is mixed evidence about the topic. In general, patients with SDHB mutation or MGMT promoter hypermethylation respond better to TMZ. Correlation between *MGMT* promoter methylation and overall survival has been studied intensively in patients with both glioblastoma and neuroendocrine neoplasm. Patients whose tumors carried a methylated *MGMT* promoter benefited more from TMZ treatment. Determination of the *MGMT* methylation status is recommended as a predictive biomarker of response in these patients (5, 26–28). However, in patients with MPP, due to the limited cases reported till now, it is still early to jump into a conclusion. Well-designed prospective clinical trials are needed to clarify the correlation between the level of *MGMT* promoter methylation and effectiveness of TMZ.

Two MPP patients were treated with TMZ in combination with capecitabine (patient 2 and patient 6 in Table 1) (11, 14). The combination therapy of TMZ and capecitabine has been widely used, and in recent years, it has emerged as the most promising and efficacious treatment for pancreatic neuroendocrine neoplasms. Capecitabine transforms into 5-FU in tumor tissues, and its metabolites can reduce the activity of MGMT, thus enhancing the effect of TMZ on DNA replication. Although the combination of TMZ and capecitabine is recommended for the treatment of pancreatic neuroendocrine neoplasms (29), it remains unknown whether the combination strategy works better than TMZ monotherapy in MPP patients. Prospective clinical trials are warranted.

In our patient, tumors shrank markedly after 3 cycles of TMZ therapy, suggesting it responded quickly to TMZ, which is consistent with the previous reports. In these papers, PR or partial metabolic response (PMR) usually occurs within 3–5 months following TMZ therapy. In the 23 patients, 12 patients and 4 patients took the drug for more than 1 and 2 years, respectively, for a maximum time period of 30 months, and some patients continued to take the drug at the end of the study (10–15). The long-term tolerability of TMZ monotherapy may be better than that of CVD therapy. Severe TMZ-related side effects are uncommon. Only 4 out of the 23 (17.4%) patients experienced Grade 3 toxicities, including lymphopenia (3 cases) and hypertension (1 case) (10–15).

CVD is the most active chemotherapy regimen for the treatment of MPP. A meta-analysis involving 50 MPP patients showed that the effective rate of CVD, in terms of tumor volume, was 55% (7). A recent retrospective study showed that, in 12 MPP patients with *SDHB* gene mutation, all patients had tumor reduction (12–100% by RECIST) upon CVD (6), which suggests CVD therapy probably works better in patients with *SDHB* mutation. However, CVD is not effective in all patients with *SDHB* mutation (patient 1 and patient 2 in Table 1). Their condition aggravated after CVD therapy but responded well to subsequent TMZ treatment. Due to the lack of prospective randomized clinical trials that compare the effects of CVD and TMZ, it is not sure which strategy is better. Previous studies seemed to show that TMZ was at least as effective as CVD regimen, and superior to CVD in that it has less side effects and is more convenient to use. In 2019, NCCN recommended both CVD regimen and TMZ for the treatment of MPP (5).

## Conclusions

We present here a MPP patient who responded remarkably well to TMZ therapy. Globally, only 26 cases of MPP treated with TMZ have been described so far. Overall, TMZ is effective and safe in patients with MPP, and is more effective in patients with *SDHB*-related MPP. Measurement of MGMT expression might help assess the tumor sensitivity to TMZ but this still needs to be systematically investigated. Whether patients without *MGMT* methylation could also benefit from TMZ therapy remains unclear and should be further explored.

## Data Availability Statement

The raw data supporting the conclusions of this article will be made available by the authors, without undue reservation, to any qualified researcher.

## Ethics Statement

Written informed consent was obtained for the publication of any potentially identifiable images or data included in this article.

## Author Contributions

AT is the first author for this case report, and she contributed to the concept and design for the study. YL is the corresponding author supervising this work. ML, YC, XM, and HW contributed to the manuscript preparation. All authors contributed to manuscript revision, read, and approved the submitted version.

### Conflict of Interest

The authors declare that the research was conducted in the absence of any commercial or financial relationships that could be construed as a potential conflict of interest.
