# Supplementary material for: Temozolomide Is a Potential Therapeutic Tool for Patients With Metastatic Pheochromocytoma/Paraganglioma—Case Report and Review of the Literature
Source: Front Endocrinol (Lausanne). 2020 Feb 18;11:61. doi: 10.3389/fendo.2020.00061 (PMC7040234; doi:10.3389/fendo.2020.00061)
Supplement: Supplementary file 1 [file Data_Sheet_1.docx]

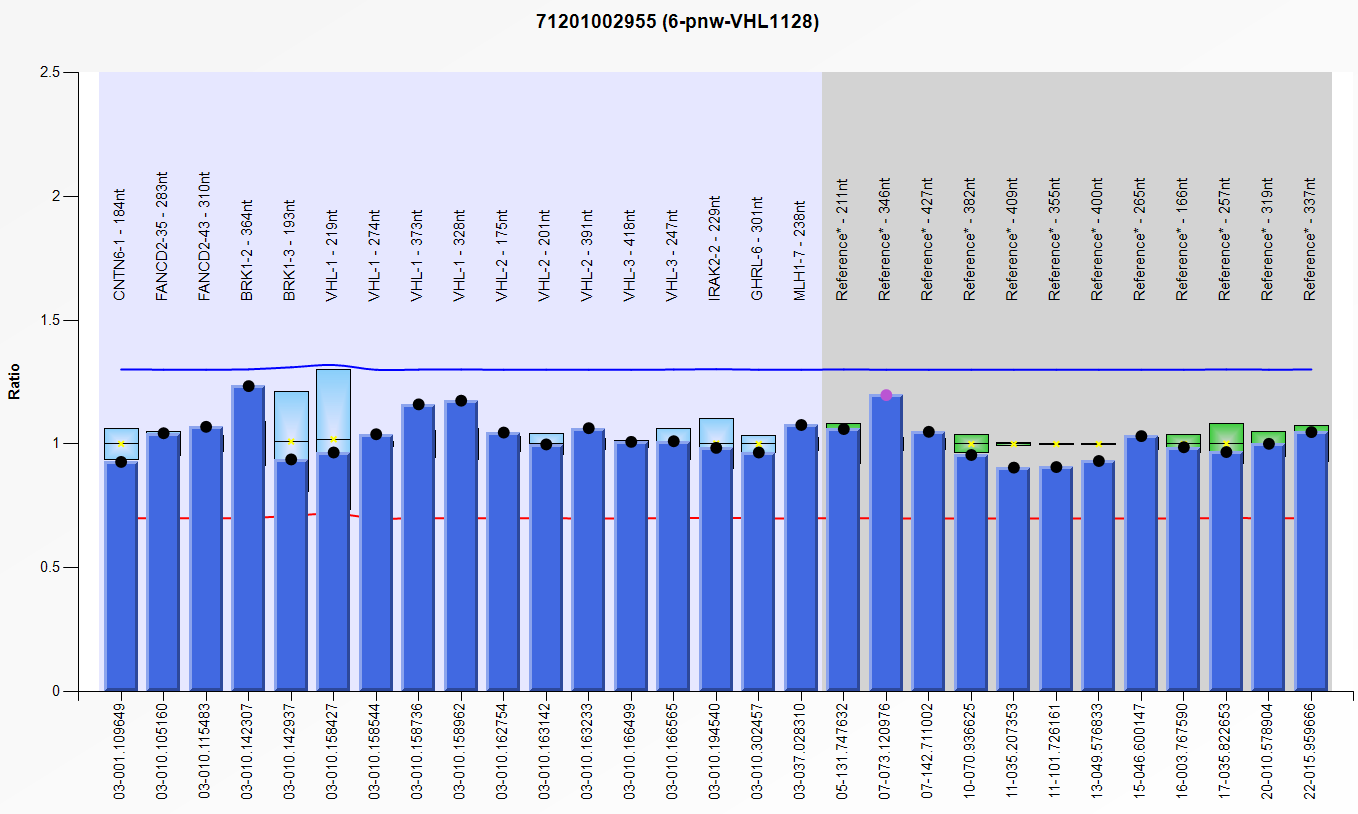


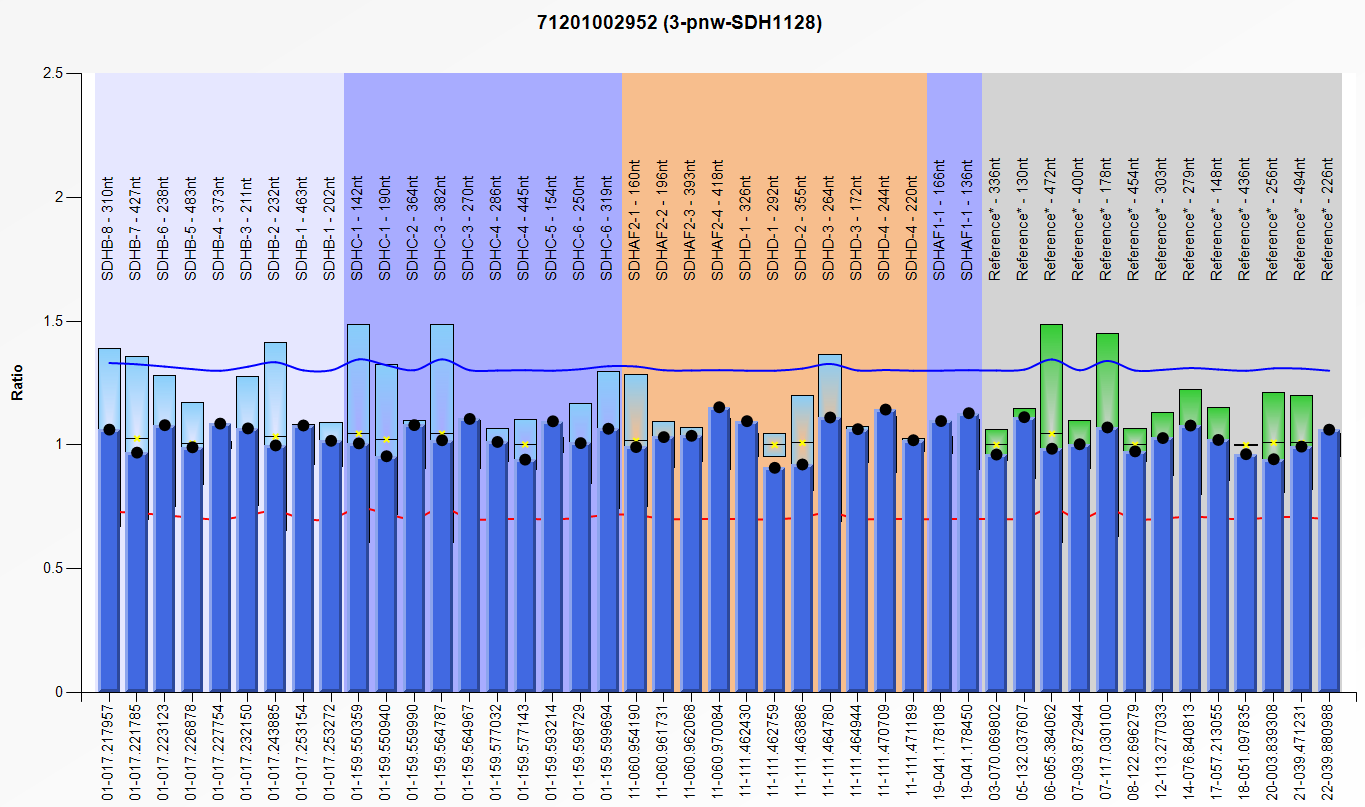


Figure S1. No large deletion of *SDHx* and *VHL* was detected by MLPA.


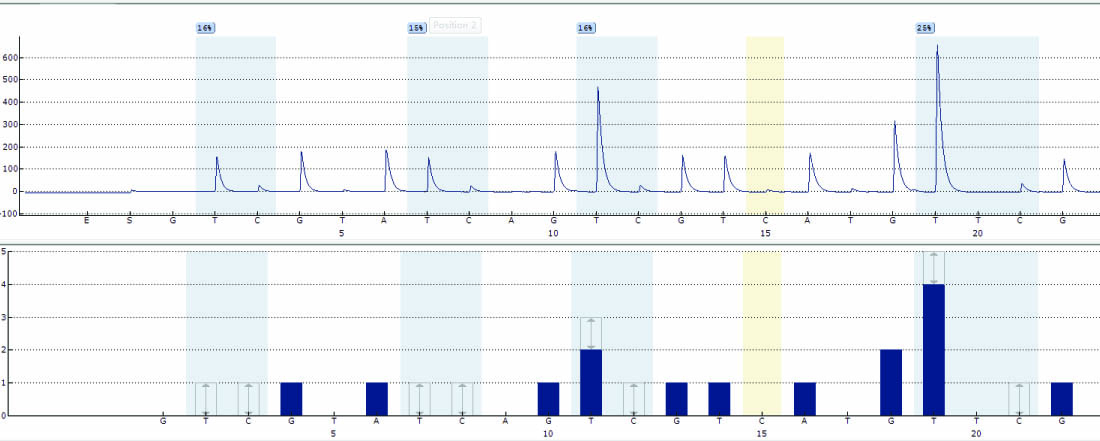


Figure S2. Methylation of four CpG sites in exon 1 of the human ***MGMT*** gene quantitatively determined. The methylation rate of *MGMT* promoter was 18%.
